# Livelihood vulnerability index: Assessment of climatic changes in flood affected areas of Mianwali district, Punjab, Pakitan

**DOI:** 10.1371/journal.pone.0315398

**Published:** 2025-03-18

**Authors:** Afshin Akram, Arifa Tahir, Asifa Alam, Anum Waheed

**Affiliations:** 1 Department of Environmental Sciences, Lahore College for Women University (LCWU), Lahore, Pakistan; 2 Global Studies Department, IGHS, Government College University, Lahore, Pakistan; University of Agriculture Faisalabad, PAKISTAN

## Abstract

The world is facing substantial threats from climate change such as extreme weather events, floods, biodiversity loss, sea-level rise, and ecosystem degradation. The objective of this study is to assess the livelihood vulnerability index of communities in flood-prone areas, specifically investigating the reasons behind their vulnerability, their income sources, and the impact of flooding on economic activities. Pakistan is an agrarian country and known to be a climate vulnerable country, flood possess higher threat to rural livelihood. Mianwali district of Punjab, Pakistan was selected as the study area because it is among the poorest districts, and is most severely impacted district during 2010 catastrophe. The study employed qualitative methods like focus group discussions, interviews, and transect walks, alongside quantitative approaches such as the Livelihood Vulnerability Index (LVI). Primary data was collected from 200 households through structured questionnaires by applying simple random sampling technique. Results demonstrated that the overall vulnerability of the local community of Mianwali to flood was high 0.4 score (out of 5) as per Livelihood Vulnerability Index criteria. It was revealed that the exposure factor (0.424) significantly influenced vulnerability and communities had low adaptive capacity (0.077) in the face of flood hazard. While the sensitivity of Mianwali’s community to flood scored 0.088. These findings are crucial for policymakers to assess baseline causes of livelihood vulnerability and formulate adaptation for other rural communities. It can be concluded that addressing these factors effectively in planning strategies may mitigate vulnerability and increase communities’ capacity to deal with potential flooding.

## Introduction

Climate change is a global and urgent concern that impacts our planet. The impact of climate change is already being felt by different countries across the world. Since the earth’s creation, climate has been changing, which makes it an inevitable phenomenon. On the other hand, climate change occurs in nature at a fairly low pace, giving species ample time to adapt to the changing environment. According to Ayanlade et al. if anthropogenic activities accelerate the change, it will happen more rapidly and species won’t be able to adapt to such rapid changes. As a result, a society has a negative impact on patterns of living, survival, and development; in developing nations, this is particularly true in the poor rural areas [1].

More frequent, intense, and extreme weather and climate-related events including warmer temperatures, sea-level rise, high rainfall, humidity, floods, damage property, critical infrastructure, impact human health, agricultural, forestry, fisheries productivity, and tourism [2]. Climate change also causes loss of ecosystems, cultural heritages and communication systems [3].

Human activity has been linked to climate change, particularly the rise in greenhouse gas emissions that occurred after the middle of the 20th century. Extreme events and other calamities are occurring more frequently, which is one of the many hazards brought on by climate change. Since AR5 (Fifth Assessment Report), there has been more evidence of observed increases in extremes such as extreme precipitation, heatwaves, tropical cyclones, and droughts, and, in particular, the attribution of these extremes to human activity. The infrastructure of society is become increasingly susceptible to extreme weather events, which are made worse by climate change [4].

It is expected that future climate change would further disrupt many areas of life, aggravating the existing challenges to prosperity posed by aged and deteriorating infrastructure, stressed ecosystems, and economic inequality. Both developed and developing countries are significantly affected by different climate hazards. Water stress and flooding vulnerability are predicted to increase due to climate change during this century [3]. Due to the rising temperature and erratic rainfall trend the resulting unfavorable floods and droughts are reinforcing higher threats to livelihood of locals [5].

South Asia is the most vulnerable region of the natural disasters in the world [6]. As a developing country, Pakistan is one of the most disaster-prone countries with flooding, earthquakes, landslides, cyclones, and other natural disasters. According to a survey more than 40% of Pakistan’s landmasses are vulnerable to flooding cyclone susceptibility, earthquakes to flooding is 60 percent and to drought is 25 percent [7]. The area has suffered enormous losses in terms of both human life and economic resources. It is predicted that in the upcoming years, the intensity, duration, and frequency of the climate change-related disasters will rise [8]. Pakistan is one of the countries with the biggest number of people directly affected by flooding each year which mainly occurs as a result of storm systems that originate in the Bengal Bay during the months of July and September. Storms from the Bay of Bengal move across lower Central India and Rajputana reaching Pakistan and continuing north via Kashmir. The mountain ranges in Pakistan’s far north provide a seasonal source of river inflow. Punjab and Sindh have been hit by floods while hill torrents look to be affecting Khyber Pakhtunkhwa’s hilly districts, Balochistan and Gilgit Baltistan. Sindh, Kabul and Swat are rivers which are prone to hazards. Pakistan continues to face flooding every year because of its climate and ecosystem. Nonetheless, once river levels rise above the average amount of flooding, these floods become dangerous. Previous floods have resulted in several deaths and considerable damage to the nation’s infrastructure [9]. The long-term temperature trends analysis shows a tendency of increase in temperature in the area. According to future forecasts, Punjab’s average annual temperature is expected to rise above the global average by the year 2100 [10]. The data in literature shows that the north of Pakistan is experiencing a more noticeable increase in temperature than the south. Rising temperatures in these northern regions have caused the Himalayan, Hindu Kush, and Karakoram glaciers to melt, creating more than 3,000 glacial lakes. 33 of them are thought to be vulnerable to floods caused by glacial lake outbursts [11].

Vulnerability assessment is the prior tool to take appropriate disaster management program in natural disaster-prone areas. The effects of climate vulnerability and related factors differ depending on the communities’ socioeconomic status, geographical location, and time frame. Accordingly, vulnerability assessment aids in determining a community’s degree of adaptability, which is the first stage in creating plans using adaptive strategies to lower the risks related to the dangers brought on by climate change [12].

These days, research on climate change places a special emphasis on how households perceive the phenomenon in order to comprehend it better. In this regard, primary data on the social, economic, and demographic profile (age, sex, occupation, income, food consumption, health, etc.) of various households are gathered. Following this, structured questionnaire surveys and focus groups are used to identify people’s perceptions of climatic fluctuations (temperature changes, rainfall, and the intensity of natural hazards). A number of techniques are also taken into consideration for assessing climate vulnerability, which looks at vulnerability across time and space and offers an opportunity to develop community-based mitigation and adaptation strategies for the future resilience [12].

Several frameworks have been proposed for assessing vulnerability, each with its own set of indicators and methods. Notable frameworks include: **Sustainable Livelihood Framework (SLF)**: This framework provides a comprehensive set of indicators that unify the often inconsistent approaches to vulnerability assessment. It focuses on the assets, capabilities, and strategies that communities use to cope with stresses such as environmental hazards [13]. **Composite Vulnerability Index (CVI)**: The CVI measures vulnerability through a set of weighted indicators that consider both external economic factors and environmental hazards. This approach enables a more nuanced understanding of vulnerability, factoring in both social and ecological components. **Social Vulnerability Index (SoVI)**: Developed to assess hazard vulnerability in U.S. counties, the SoVI evaluates a range of socioeconomic and demographic variables (e.g., income, education, race, and age) to measure a community’s ability to respond to and recover from natural disasters [14]. This index highlights how social structures can influence vulnerability to climate-related risks.

In addition to these frameworks, **indicator-based numerical approaches** such as the Livelihood Vulnerability Index (LVI), developed by Hahn et al. [8], and the IPCC Vulnerability Index [9], which incorporates the three key dimensions of vulnerability—exposure, sensitivity, and adaptive capacity—are widely used in the literature to assess the vulnerability of communities to climate hazards. These indices are instrumental in assessing the vulnerability of a region and its adaptive capacity in the face of natural disasters. The LVI, for instance, evaluates how various factors, including economic, social, and environmental components, interact to determine a community’s vulnerability to climate-induced stressors [15].

**Remote sensing technologies** have also become crucial in monitoring climate-related hazards. Satellite observations have revolutionized our ability to track and manage natural disasters, including droughts, floods, and food shortages. By providing real-time data on land use and land cover changes (LULC), remote sensing tools help in mapping the vulnerability of affected areas. Techniques like the Standardized Precipitation Index (SPI) are increasingly used to identify patterns of wet and dry seasons, helping in the development of early warning systems for communities at risk of climate-induced disasters (National Drought Monitoring Centre, 2018) [16].

While exposure to hazards may be similar across different communities, the **impacts** of these hazards often depend on each community’s ability to cope with and manage these risks. Vulnerability assessments, therefore, play a critical role in understanding how different communities are affected by natural hazards and in designing targeted interventions to reduce their vulnerability [17–19].

Understanding the relationship between **climate projections**, societal structures, political systems, and health outcomes is crucial to addressing the complexities of climate change. Many vulnerability frameworks in the literature have focused on sensitivity and adaptive capacity, providing valuable insights into how communities may fare in the face of changing climate patterns [20–24].Previous vulnerability assessment framework includes sustainable livelihood framework, composite vulnerability index and social vulnerability index. The sustainable livelihoods framework provides a unified framework of indicators for the current inconsistent classification of vulnerability assessment index [13]. Composite Vulnerability Index evaluates external economic factors and environmental hazards to measure vulnerability by means of a set of weighted indicators previously selected. Hazard vulnerability in US counties is examined by the Social Vulnerability Index (SoVI) through the assessment of 11 variables related to socio-economic and demographic dimensions [14].

In this background, the present study aimed to evaluate the vulnerability level in study area, identify the weak aspects of the community and people’s livelihood assets to adapt with the natural hazards using different indices and natural capitals and to assess which factors contribute more towards the vulnerability of households in the face of changing climate. The goal of the study was to gain a better understanding of people’s livelihoods and vulnerability in flood-prone areas and to analyze how socioeconomic and environmental changes affect the livelihood, susceptibility, and adaptive capacity of different social classes. The current study investigated the flood’s social and economic vulnerabilities, as well as the effects it had on people and their belongings. For this purpose, focus group discussions along with other qualitative techniques were used carried out. There are few studies undertaken in Mianwali that provided a foundation for understanding how people’s livelihoods were affected make individuals more vulnerable.

## Materials and methodology

### Study area

**Fig 1:**
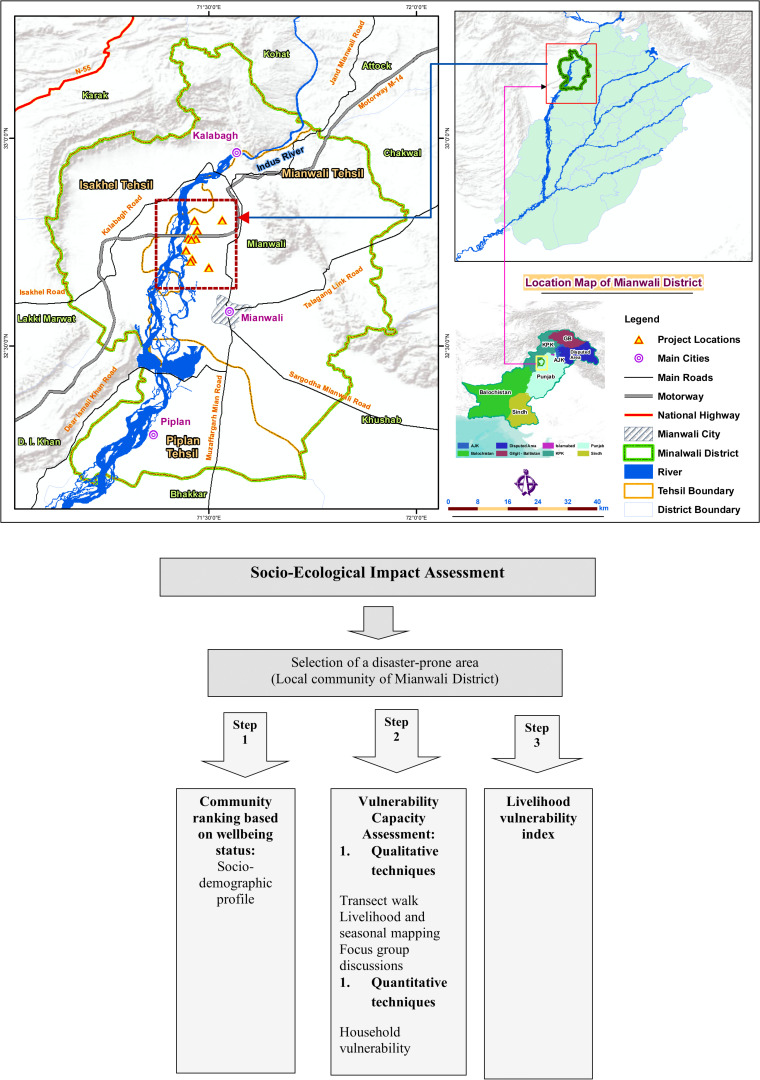
A: Map of the study area. B: Research methodology.

In the North West of the Punjab province, District Mianwali is located representing the western plains of the salt-ranges close to the Sakesar hill [25]. Geographically district Mianwali lies between 32° 30′ and 33° 14′ N. and 71° 7′ and 71° 44′ E., covering an area of 5840 square kilometers (Fig 1A) . There are three sub-districts in Mianwali: the northern and western part of the district is occupied by Isa Khel, Mianwali lies in the east, and Piplan covers the south [26]. Climate of the district is severe with cold winters with little rain and extended hot summer. June and July are the hottest months with maximum temperature of 48° C whereas December and January are the coldest months with minimum temperature of 4-5 ^0^C. During the monsoon season, this district is traditionally vulnerable to floods and overflow. According to PPA, Mianwali is a poor district ranking 29^th^ out of 36 districts on the Multi-Dimensional Poverty Index [26].

### Research design explanation

Our study employs a mixed-methods research design, integrating both qualitative and quantitative approaches. In this mixed method approach, the quantitative and qualitative data is combined to develop descriptive and subjective measures and the information obtained through the two approaches is then triangulated to interpret the results [25]. This combination allows for a comprehensive analysis of livelihood vulnerability in flood-prone areas by capturing both numerical data and in-depth personal insights. The qualitative methods help in understanding the nuanced impacts of floods on local communities, while the quantitative methods provide measurable data to support our findings.

### Data collection

Baseline data such as socio-economic indicators, flood history in the district and potential hazards of the district was collected from District Disaster Management Authority (DDMA). Three phases of the study process were carried out, one for each of the objectives, in order to accomplish them. In order to collect the data, the basic stages were the establishment of direct communication with the community and its residents and then evaluate official sources of information. In order to categorize the community into well-being groups, a total of 200 households were randomly surveyed to fill the questionnaire as followed by Shahzad [25]. Data was collected in April 2021 and was accessed in December 2021. As Urdu was the language widely spoken in the local community, interview questions were asked in Urdu language and questionnaires were translated in Urdu as well. For the collection of data the risk analysis data provided in the Table 3 was taken into consideration.

**Table 3:** Characteristics of population indicating well-being categories.

Indicators of well-being	Categories			
	Well-off (20)	Better-off (40)	Poor (80)	Very poor (60)
Education	Children were attending private schools, colleges and universities/ girls were getting higher education. (30%)	Children were going to private and public schools. (25%)	Their children were attending public schools. (25%)	Children were involved in labor. (20%)
Ownership of land	They were living in their own house and had large productive land. (20%)	They had a house smaller than well-off families & small agricultural land. (27%)	They were living in rented house or in huts. (23%)	They had no land for house and were living in temporary huts. (30%)
Food	They had sufficient food storage & access to big food markets. (10%)	They grow food enough for their family. Store food for 3 months. (22%)	They had to buy food products on daily basis. (28%)	They had no storage of food. (40%)
Employment (livelihood)	More than one person in home had job in big cities/doing business (15%)	Doing job in Mianwali/ involved in government jobs (10%)	They were doing labor on daily wages/ involved in aquaculture. (40%)	Mostly unemployed/ involved in labor (35%)
**Indicators**	**Well-off**	**Better-off**	**Poor**	**Very poor**
Health condition	Healthy members mostly/ they had filtered water source in home. (10%)	Healthy members with some acute illness/ clean drinking water supply (25%)	They had to collect water from far-off sources/ at least 1 member suffering chronic illness (25%)	No access to clean drinking water/ more than 2 individuals was diseased. (40%)
Dependence on natural resources	They had sui gas supply in their homes. (20%)	Gas cylinders in their homes/ collect firewood on weekly basis. Raising livestock (20%)	They collect and sell firewood. And also had pets (hen, cow). Also depend on fishing. (40%)	Collection and selling of firewood. (20%)
Migration pattern	One or more family member was working abroad (10%)	Domestic migration for a week or less (jobs in nearby city or town) (70%)	Migration for wage labor (40%)	N\A

The following aspects have been considered for participant confidentiality and ethical consideration in this research: First, anonymity is ensured by using unique identifiers instead of names. Second, data protection measures are implemented, including encrypted files and limited access for authorized personnel. Third, informed consent is prioritized, with participants clearly informed about data usage and storage, allowing them the option to withdraw at any time. Additionally, data will be securely disposed of after the study, with digital files deleted and physical documents shredded. Finally, results will be reported in a way that prevents the identification of individual participants, such as through aggregation or generalization. These measures uphold ethical standards and create a safe environment for all participants.

### Qualitative methods

1. **Transects walks: **Researchers walked through the community with local guides to observe and record environmental and infrastructural conditions, capturing real-time data on flood impacts and community responses.
2. **Focus Group Discussions:** In this study, Focus Group Discussions were conducted with different segments of the community, including farmers, women, and local leaders. The discussions aimed to gather diverse perspectives on how floods affect various aspects of life and livelihood.
3. **Individual Interviews**: Semi-structured interviews were carried out with key informants and household heads to gain detailed personal accounts and experiences related to flood impacts.

### Quantitative methods

#### Structured questionnaires.

The study utilized a structured questionnaire administered to 200 households, selected to provide a comprehensive representation of the community’s socio-economic status, livelihood assets, and adaptive capacity (Questionnaire is given in Supporting Information 3). The sample size of 200 was determined based on several factors: first, it was sufficient to ensure statistical reliability and allow for meaningful comparisons across various demographic groups within the community. Second, this number strikes a balance between being manageable for data collection and providing a robust dataset that can capture the diversity of experiences and vulnerabilities present in the population.

To ensure the sample’s representativeness, households were selected using a stratified sampling approach, which involved categorizing the community based on key characteristics such as income levels, geographic location, and reliance on different livelihood sources. This method allowed the study to capture a wide range of perspectives and experiences related to flood vulnerability. The questionnaires were carefully designed to collect consistent and comparable data across all surveyed households, enabling a thorough analysis of the factors influencing community vulnerability

#### Software package used.

For the analysis of the collected data, we utilized the SPSS (Statistical Package for the Social Sciences). This software was used for the quantitative data analysis. It facilitated the statistical analysis of survey data, allowing us to identify patterns, correlations, and significant factors contributing to livelihood vulnerability.

The LVI was calculated using a structured approach that involved multiple steps to ensure a robust assessment of livelihood vulnerability. Each major component was derived from specific sub-components, which were assigned weights based on their relevance and contribution to overall vulnerability. Weights for each sub-component were determined through expert consultations and a review of relevant literature, ensuring that the most significant factors influencing vulnerability were adequately represented. The normalization process involved min-max normalization, allowing for a balanced comparison of diverse indicators. We standardized the values of each sub-component by transforming the data to a scale of 0 to 1. This approach facilitates comparison among different indicators without distortion and ensures that all sub-components contribute proportionately to the final LVI score.

#### Integration of methods.

The mixed-methods approach provided a robust framework for our research. By integrating qualitative insights with quantitative data, we were able to cross-validate findings and provide a more comprehensive understanding of the livelihood vulnerabilities faced by communities in flood-prone areas. The qualitative data enriched the quantitative findings with context and depth, while the quantitative data provided a broader perspective and statistical validation of observed patterns. Details of research methodology followed are shown in Fig 1B.

### Community ranking based on well-being status

A participatory approach of community livelihood based on their wellbeing status was used to rank the community on criteria of wealth and poverty. This approach was developed by Pakistan Participatory Assessment for the assessment of the level of relative wealth and poverty among the locals. To classify the surveyed population into wellbeing categories, the basic characteristics of population were asked. Respondents were questioned regarding their possession of land as owning land was considered a sign of richness and stability in that area. In addition, respondents were questioned whether their children attend private or government schools. Access to food and availability of local resource were also asked. They were also asked about their health status as to whether people had chronic disease (more than 3 months) or acute disease (a week or more) and migration patterns were also asked.

Local community was categorized into four well-being groups named as well-off, better off, poor and very poor people based on the data collected. For empirical validity, scholars have broadly used well-being categorization based on socio-economic status [27–29].

#### Vulnerability capacity assessment of local community.

In this study a combination of various participatory and qualitative methods was used to gather data from residents as community representatives, NGO employees and government organizations. The technique of Participatory rural appraisal (PRA) was used as this technique was used in many previous studies [25]. The participatory tools used in this study include Focus Group discussions (ranking of hazard severity, livelihood and seasonal mapping, institutional strength) and Transect Walk [30,31]. In the community of Mianwali, a transect walk was done to determine resource distribution and location. Because it provided good description of land usage, resource forms and uses, vegetation, everyday supply of water, and living conditions etc. Per walk, 10 people were questioned and the data obtained during the walk was stated on sheets of paper. Focus Group Discussions (FDGs) were conducted with ten people for each discussion, ensuring diversity in terms of age, gender, religion, race, and caste. The FGDs took place in nearby small rest houses, with a total of four discussions involving 40 participants (10 females and 30 males). The FGDs aimed to acquaint stakeholders with the different indicators used to evaluate vulnerability in the VCA method, to collect the data available from the community and to familiarize stakeholders with significant vulnerabilities and approaches (solutions) to mitigate the vulnerabilities. The respondents were asked about the impacts of flooding on their livelihoods, including its effects on food availability, water quality, access, and agricultural production. The study also investigated the current availability and seasonal variations of resources, identifying any resources that were previously present but no longer available. The participants were questioned about their flood-protection systems and the natural resources they rely on for their livelihoods and daily activities. Informants were asked about their methods of storing food and water, and community mobility was explored in relation to migration patterns from Mianwali district to larger cities for better livelihood opportunities or other facilities. The severity of the hazards that significantly affected their livelihoods was ranked on a scale of 0–5, with 5 indicating the highest level of hazard. Each household representative was also asked about their coping strategies. Finally, the vulnerability status of different social classes was determined based on the available livelihood options in the region. This comprehensive data collection process allowed for the creation of livelihood and seasonal mapping, as well as the ranking of hazard severity.

#### Livelihood vulnerability quantification.

An index namely Livelihood Vulnerability Index was used for the quantification of livelihood vulnerability from floods in the study area. The idea of using an index for vulnerability assessment was derived from the basic requirement of vulnerability by Intergovernmental Panel for Climate Change (IPCC). Two approaches used for this purpose were: LVI and LVI-IPCC. Livelihood Vulnerability Index or LVI was developed by Hahn [8], Madhuri [32], Simane [33] and Richardson [34]. The Livelihood Vulnerability Index was based on the descriptive information gathered from the field survey which was based on 41 sub-components [19]. These subcomponents were combined into 8 major components and three vulnerability factors. The detail of components and subcomponents of vulnerability is shown in Table 1:

**Table 1:** Key Components and Sub-Components of the Livelihood Vulnerability Index (LVI) used to assess Vulnerability.

Vulnerability factors/ Major Components	Sub-components	Surveyed questions
**Adaptive Capacity**	1. **Socio-Demographic profile**	
	% of HH having female as head % of HH have access to TV and radio % of HH having an educated head	How many households have a female as the head? How many households have access to both TV and radio? How many households have an educated head?
	2. **Livelihood strategies**	
	% of HH working in government offices % of HH working outside the city % of HH having livelihood without any contribution of cultivation of crops % of HH having aquaculture (fishing) main source of livelihood % of HH having agriculture main source of livelihood % of HH having no direct water supply facility to produce crops % of HH having their own land which is used for agriculture	What percentage of households have at least one member working in government offices? What percentage of households have at least one member working outside the city? How many households have a livelihood that does not involve the cultivation of crops? How many households have aquaculture (fishing) as their main source of livelihood? How many households have agriculture as their main source of livelihood? How many households do not have a direct water supply facility for crop production? How many households own land that is used for agriculture?
	3. **Social networks**	
	% of HH having health insurance or free health care % of HH gets at least one of the financial loan in the last 12 months % of HH seeking help from any government office % of Presence of any private money lender % of HH having social support	1. How many households have health insurance or access to free healthcare? 2. What percentage of households had at least one family member who obtained a financial loan in the last 12 months? 3. How many households sought help from any government office? 4. Is there any presence of private money lenders in your area for borrowing money? 5. How many households have access to social support? .
Sensitivity	**Health status of members in household**	
	% of Health center in your area % of HH having member with chronic illness % of Affordability of basic health (emergency) services % of Accessibility to basic health facilities % of HH using wood as a fuel for cooking food % of HH having toilet in their house	How many health centers are available in your area? What percentage of households have at least one member with a chronic illness? What is the level of affordability of basic health (emergency) services for households? How accessible are basic health facilities for households in your area? What percentage of households use wood as a fuel for cooking food? How many households have a toilet in their house?
	**Food related issues**	
	% of Food sufficiency % of HH dependency on fishing for food % of HH primarily dependent on self farmed food % of adequacy of access to food markets % of store seeds (grain)	What percentage of households have food storage that can sustain them for at least 6 months? How many households depend on fishing as their primary source of food? How many households primarily rely on self-farmed food for their sustenance? How adequate is the access to food markets in terms of roads and market availability? How many households store seeds, specifically grains, for future use?
	**Water related Issues**	
	% of accessibility to clean drinking water % of HH having pipe supply of water % of reliance on natural water resources % of Water from the main pipe supply drinkable	What is the level of accessibility to clean drinking water in the area? How many households have a pipe supply of water? To what extent do households rely on natural water resources for their water supply? Is the water from the main pipe supply considered drinkable
**Exposure**	**Natural disaster**	
	% of floods in the last 5 years % of family members receive any flood warning before the flood % of Damage to physical property and agriculture by the flood % of an immediate family member physically or emotionally injured in the flood % of any flood warning agency in your area % of affected individuals require hospitalization % of people shifted because of flood destruction % of HH whose house disconnected due to flood	What is the historical trend of floods in the area over the past 5 years? How effective are flood warning systems in alerting residents before a flood occurs? What is the extent of damage caused to physical property and agriculture as a result of floods? Have there been instances of immediate family members being physically or emotionally injured during floods? Are there any flood warning agencies or organizations operating in the area? What is the percentage of affected individuals who require hospitalization due to flood-related incidents? Has there been a significant number of people who had to be relocated due to flood destruction? What percentage of households experienced disconnection from the main road as a result of flooding?
	**Climatic Variability**	
	Monthly average of maximum daily temperature (Celsius) Monthly average of average minimum daily temperature (Celsius) Monthly average precipitation (Milimeters)	Mean standard deviation of monthly average of maximum daily temperature Mean standard deviation of monthly average of minimum daily temperature Mean standard deviation of monthly average precipitation?

#### Approach 1-Livelihood Vulnerability Index (LVI) calculation.

- Based on the field survey questions, 8 major components were queried. These major components were calculated at household level namely socio-demographic profile (SDP), Livelihood strategies (LS), Social network (SN), health (H), food (F), water (W), natural disaster (ND) and climatic variability (CV). These components were inquired using 35 sub-components.
- Following four steps were used to measure each major component of LVI

1. Calculate subcomponent data from the questionnaire and convert it into percentage and index
2. Unit standardization of all transformed data was performed for each subcomponent. It was done to balance the weights based on Sullivan [35].
3. To get the final value for each main component, compute the average of each standardized value
4. The final step includes the weighted averages of all major components to get the LVI value.

This procedure affirms the equivalent contribution of major components in generating the overall LVI. As described by Hahn [19], the LVI scale calculated from this approach ranges from 0 (minimum) to 0.5 (maximum). The min-max normalization technique [36] was adopted to normalize variables.

The normalization method, which is frequently employed in the literature on climate change susceptibility, was utilized to calculate the vulnerability index [37]. We used the following formula to all indications.


$$Normalvalue=\left(ActualValue-Minimumvalue\right)/\left(MaximumValue–Min\right)$$ (1)

The formula followed is


$$index_{s}=\frac{S_{s-s_{\min}}}{s_{max-}s_{min}}$$ (2)

Where, *S*_*s*_ =  original sub-components for each of the site*;*

*Smin* **=** the minimum value for each sub-component,

*S*_*max*_ *=  the* maximum value for each sub-component.

- The average of each sub-component was determined using the formula after obtaining the values of the sub-components

$$Ms=\frac{\sum_{i=1}^{n}index_{s_{s^{i}}}}{n}$$ (3)

M_S_ = one of the eight major components for study site

$index_{s_{s^{i}}}$ = subcomponent, n=number of sub-components in each major component.

The above values were calculated for each of the eight major components and LVI was calculated using the following formula


$$LVI_{s}=\frac{w_{SDP}SDP_{S}+w_{LS}LS_{S}+w_{SN}SN_{S}+w_{H}H_{S}+w_{F}F_{S}+w_{W}W_{S}+w_{CV}CV_{S}+w_{ND}ND_{S}}{w_{SDP}+w_{LS}+w_{SN}+w_{H}+w_{F}+w_{W}+w_{CV}+w_{ND}}$$ (4)

Where SDP denote socio-demographic profile, LS refers to livelihood strategies, SN stand for social network, F represent food, W correspond to water, H symbolize health, CV represent climatic variability and ND stand for natural disaster. w =  weighting factor for each component.

#### Approach 2-IPCC framework for calculating LVI.

An alternative approach was also used to measure the LVI which integrates the IPCC definition of vulnerability. The LVI–IPCC framework organizes the eight major components that were used in the first approach. Table 1 demonstrates the organization of those components into the LVI-IPCC framework. According to the framework [36,38], Exposure is symbolized by e and it is measured by the number of natural disasters that have happened over the past 5 years, while the climate variability is measured by the average standard deviation of the maximum and minimum monthly temperatures and monthly precipitation over a span of 9 years. Adaptive capacity is assessed by the district’s socio-demographic profile, the livelihood strategies used and the power of social networks. Moreover, vulnerability is determined by assessing the existing condition of food and water safety and health status in a region.


$$LVI-IPCC_{s}=\left(e_{s}-a_{s}\right)\times s_{s}$$ (5)

where LVI–IPCC_s_ is the LVI for district s represented by using the IPCC vulnerability framework, e_s_ is the computed exposure score for district s (equivalent to the Natural Disaster and Climate Variability major component), a_s_ is the calculated score of adaptive capacity for district s (weighted average of the Socio-Demographic, Livelihood Strategies, and Social Networks major components), and s_s_ is the calculated score of sensitivity for district s (weighted average of the Heath, Food, and Water major components). The scale of LVI–IPCC ranges from -1 (least vulnerable) to + 1 (most vulnerable).

## Results

### Baseline data of study area

Socio-economic indicators such as population, number of Tehsils and Union Council, number of Revenue estates, population density, family size, population growth rate, poverty rate, population/doctor ratio, infant mortality ratio, maternal mortality ratio, literacy rate, Global Acute Malnutrition (GAM) rate, Severe Acute Malnutrition (SAM) rate, population having access to clean water, annual crime rate of Mianwali has been summarized in the Table 2 below;

**Table 2:** Socio-economic indicators of Mianwali district.

Sr. #	Socio-Economic Indicators	Numbers
1)	Population	1,546,O94
2)	No. of Tehsils &UCs	3 & 51
3)	No. of Revenue Estates	256
4)	Population Density (people per km2)	264.70
5)	Average Family Size	4
6)	Population Growth Rate	2.02
7)	Poverty Rate	46%
8)	Population/Doctor Ratio	1000/1
9)	Infant Mortality Rtae (IMR)	77/1000 live births
10)	Maternal Mortality Ratio (MMR)	140/100,000 live births
11)	Literacy Rate	62%
12)	Global Acute Malnutrition (GAM) rate	17.7%
13)	Severe Acute Malnutrition (SAM) rate	6.4%
14)	Percentage of population having access to clean drinking water	90%
15)	Annual Crime (Punjab Development Statistics)	5552

### Wellbeing status of the study population

The survey of the Mianwali district for data collection was conducted to know the well-being status and livelihood trends of locals resulted in categorization of community. The characteristics of population indicating wellbeing categories is given in Table 3. For the 200 households studied, 13 percent (n = 26) had their own home and farm land, while 32.5% (n = 65) stayed in leased houses and operated on leased farms. On contrary, 44.5% (n = 89) of citizens work in property belonging to others. 10 percent (n = 20) of the total households surveyed were very poor as they were living in temporary huts or kacha houses. People living in a house constructed of mud and chaffed roof, if moist, dampened by flood water and making it prone to collapse. Well-off residents had cemented homes that are resistant to floods and away from banks of the river. On the basis of “ownership of land” criterion, 13% people were categorized as well-off, 32.5% were better-off, 44.5% were poor and 10% households were very poor. Earlier floods threatened the livelihoods of all four income classes but poor and very poor citizens were particularly at high risk because they had no protective measures or proposals to save their homes or employment etc.

Recurring floods are destroying their agricultural property, which is the principal source of livelihood for the majority of the people, according to the local community viewpoint. Almost all better-off families retained grain stock for at least 3 months. Poor households had limited means of income as well as less production of food. Because of the floods, crop destruction had a major effect on poor people’s food supply. Flood was considered a threat among all well-being groups. In terms of education, the households with higher qualifications had a range of income generating opportunities. In households listed as well-off, their children got higher education in colleges and universities in big cities like Lahore. Children of poor families attended public (government) schools. Kids from very poor families were involved in forced labor (such as serving in the home of wealthy people. As for the trend of migration, well-off households have members working abroad or in Pakistan’s big cities. While better-off families had migrated seasonally to some other town or area. It was reported that most poor families migrate seasonally or regularly for wage labor, or sell their agricultural/ livestock products.

### Livelihood vulnerability index

Vulnerability was measured at household level and findings are described in Table 4. Based on the data obtained and observed Mianwali was found to be vulnerable to repeated floods with calculated numerical value of 0.4. This high value shows that the community residing along riversides was more vulnerable. The component health issue had highest scores (0.58) than the other major components. The score of food availability component was 0.49. Most of the households were dependent on the self- farmed food. For water availability in households, Mianwali had an index value of 0.255. Some of the households were more reliant on natural sources of water to fulfill their everyday water needs. Socio-demographic profile had an index value of 0.54. Most of the households had T.V and radio in their homes. Many households had a male head and their qualification was middle or matric. The local population was more vulnerable to the flooding, because the natural hazard exposure component had a score of 0.34. Also, the climate variability score (0.127) was determined using the Mianwali meteorological data. The value of livelihood strategy index is 0.38. This indicates that family dependent on the agricultural sector is more vulnerable to flooding than those families who rely not just on the agriculture industry. Based on the level of vulnerability classification conducted by Hahn *et al*., (2009), the scale of the LVI value is set between 0 (not vulnerable) and 0.5 (very vulnerable). Generally, the local population of Mianwali was found to be vulnerable as the aggregated value of LVI for Mianwali district is 0.4.

**Table 4:** LVI score for Mianwali.

Variability parameters	Variables	Questions inquired	Units	Mianwali			
				Actual value	Standardized value	Max. value	Min. value
Exposure	Natural disasters	Number of floods in the last 5 years?	Count (0-5)	2.8	0.27	5	02
		Families receive any flood warning before the flood	Percentage	39	0.39	100	0
		Percent of the property damaged by the flood?	Percentage	40.5	0.405	100	0
		An immediate family member physically or emotionally injured in the flood?	Percentage	25	0.25	100	0
		Any flood warning agency in your area?	Percentage	26	0.26	100	0
		Did the connection of your house to main road disconnect due to flood?	Percentage	50.5	0.505	100	0
		Affected individual require hospitalization?	Percentage	20	0.2	100	0
		You replace from your area as result of flood destruction?	Percentage	47	0.47	100	0
					0.34		
	Climatic variability	Mean standard deviation of monthly average of average maximum daily temperature?	Celsius	17.8	0.051	42.9	16.45
		Mean standard deviation of monthly average of average minimum daily temperature?	Celsius	8.6	0.192	29.2	3.7
		Mean standard deviation of monthly average precipitation?	Mm	73.3	0.138	530	0
		0.127					
Sensitivity	Health	Presence of Health center in your area?	Percentage	56.5	0.565	100	0
		Household have any with chronic illness?	Percentage	35.5	0.355	100	0
		Affordability of basic health (emergency) services	Percentage	40	0.4	100	0
		Accessibility of basic health facilities	Percentage	29.5	0.295	100	0
		Household using wood as a fuel for cooking food?	Percentage	95	0.95	100	0
		Household having toilet in use	Percentage	90	0.9	100	0
					0.58		
	Food	Food sufficiency (households have food storage for at least 6 months)?	Percentage	54.5	0.545	100	0
		Households store seeds?	Percentage	66	0.66	100	0
		Household dependency on fishing for food?	Percentage	25	0.25	100	0
		Households primarily dependent on self farmed food?	Percentage	59	0.59	100	0
		Adequacy of access to food markets (roads/markets)?	Percentage	41.5	0.415	100	0
					0.492		
	Water	Accessibility to clean drinking water?	Percentage	52	0.52	100	0
		Households having pipe supply of water?	Percentage	17.5	0.175	100	0
		Reliance of household on natural water resources (river, lake or rain)	Percentage	22.5	0.225	100	0
		Water from the main pipe supply drinkable?	Percentage	10	0.1	100	0
					0.255		
Adaptive capacity	Socio-demographic	Households having female as head	Percentage	17.5	0.175	100	0
		Households have access to TV and radio?	Percentage	77	0.77	100	0
		Households having an educated head	Percentage	67	0.67	100	0
					0.54		
	Livelihood strategies	Member of household working in government offices	Percentage	34.5	0.345	100	0
		Any member of household working outside the city?	Percentage	14.5	0.145	100	0
		Household having livelihood without any contribution of cultivation of crops	Percentage	40.5	0.405	100	0
		Household having aquaculture main source of livelihood?	Percentage	28.5	0.285	100	0
		Household having agriculture main source of livelihood?	Percentage	65	0.65	100	0
		Household having no direct water supply facility to produce crops?	Percentage	52.5	0.525	100	0
		Households having their own land use for agriculture?	Percentage	34.5	0.345	100	0
					0.38		
	Social networks	Households having health insurance or free health care?	Percentage	15.5	0.155	100	0
		Any family member got at least one source of financial loan in the last 12 months	Percentage	39	0.39	100	0
		Households seeking help from any government office?	Percentage	8.5	0.085	100	0
		Presence of any private money lender in your area to borrow money from?	Percentage	44	0.44	100	0
		Households having social support?	Percentage	6	0.06	100	0
					0.23		

## Approach 1


$$LVI_{s}=\frac{w_{SDP}SDP_{S}+w_{LS}LS_{S}+w_{SN}SN_{S}+w_{H}H_{S}+w_{F}F_{S}+w_{W}W_{S}+w_{CV}CV_{S}+w_{ND}ND_{S}}{w_{SDP}+w_{LS}+w_{SN}+w_{H}+w_{F}+w_{W}+w_{CV}+w_{ND}}$$

$$LVI_{s}=\frac{\left(0.54\times 3\right)+\left(0.38\times 7\right)+\left(0.23\times 5\right)+\left(0.58\times 6\right)+\left(0.492\times 5\right)+\left(0.255\times 4\right)+\left(0.127\times 3\right)+\left(0.34\times 8\right)}{3+7+5+6+5+4+3+8}$$

$$LVI_{s}=0.38≅0.4$$

**Fig 2:**
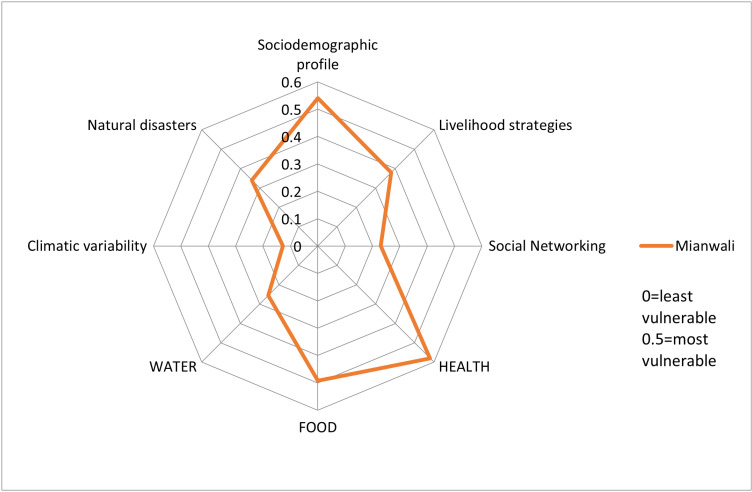
Spider diagram showing scores of major components contributing to the livelihood vulnerability index (LVI).

After calculating LVI scores for the district Mianwali, a spider diagram was created as shown in Fig 2 to show the scores of major components contributing to the livelihood vulnerability index. In the study area, health issues had comparatively higher scores than those of the other components.

### Calculation of adaptive capacity through LVI-IPCC

**Fig 3:**
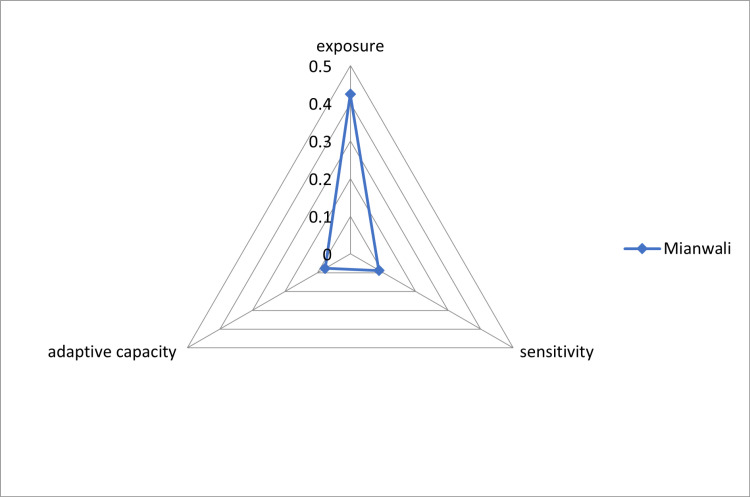
Three major components of LVI-IPCC i.e., adaptive capacity, exposure, and sensitivity regarding Mianwali.

The three major components (Adaptive capacity, Exposure and Sensitivity) that contribute to the LVI-IPCC were calculated from eight major components of LVI (Fig 3). Fig 3 shows the three major components of LVI-IPCC i.e., Adaptive capacity, exposure and sensitivity of Mianwali. The vulnerability factor “Adaptive capacity” comprised of socio-demographic profile, livelihood strategies and social network of local community. Exposure factors consist of climatic variability and natural disasters. In sensitivity index, health, food and water related issues were considered. There are three categories for LVI-IPCC as given by IPCC [17] to check the vulnerability status. The categories of LVI-IPCC overall analysis is given in Table-5 showed that the Mianwali district had very low adaptive capacity. High exposure was attributed to the recurring flooding in the study area. Results of LVI-IPCC showed that the exposure value for Mianwali community was high (0.424). This high value implied a high vulnerability to flooding and climatic variability for the community. The final IPCC weighted LVI scores range between (−1) is not vulnerable and (1) is the highly vulnerable. On the whole, LVI and LVI-IPCC for Mianwali scored 0.4 and 0.0305 respectively. LVI-IPCC results given in Table 5 indicates that the Mianwali community is vulnerable to floods.

**Table 5:** LVI and LVI-IPCC based on contributing factors and vulnerability scores for Mianwali.

Contribu ting factors	Major components	No. of sub-components	Major components values	Contributing factor values
**Adaptive capacity**	Socio-demographic	3	0.54	0.077
	Livelihood strategies	7	0.38	
	Social network	5	0.23	
**Exposure**	Climatic variability	3	0.127	0.424
	Natural disasters	8	0.34	
**Sensitivity**	Health issues	6	0.58	0.088
	Food related issues	5	0.492	
	Water issues	4	0.255	
LVI (0 minimum to 0.5 maximum) LVI-IPCC (-1 least vulnerable to + 1 most vulnerable)		0.4 LVI – IPCC_s_ = (e_s_ - a_s_) × s_s_ = (0.424 – 0.077) × 0.088 = 0.0305		

A Vulnerability triangular diagram was developed using the scores of LVI-IPCC calculated from LVI. It ranges from 0 to 0.5 demonstrating that Mianwali has a high level of exposure with low adaptive capacity. Mianwali district is geographically prone to repeated floods. People have faced many floods and consequently some adaptations have been learned by them.

### Risk analysis: potential hazards of the district

DDMA data regarding flood history in the study area depicts that in 2010, a total of 461,472 people were affected by floods, resulting in 12 deaths. Additionally, 126 revenue estates were affected, with 163,218 houses being partially damaged and 107,190 houses being fully damaged.

In 2011, there is no data available for the number of people affected, deaths, revenue estates affected, or houses damaged, indicating that there were no significant flood events during that year. Similarly, in 2012, 2014, 2016, 2017, 2018, 2019, 2020, and 2021, there were no reported flood events, as indicated by the absence of data in the respective columns.

In 2013, 64,124 people were affected by floods, but there were no reported deaths. Ten revenue estates were affected, with 12,885 houses being partially damaged and 3,206 houses being fully damaged. In 2015, 15,420 people were affected by floods, resulting in four deaths. Additionally, 95 revenue estates were affected, with 56 houses being partially damaged and 1,299 houses being fully damaged.

The historical flood data presented in the above paragraph clearly demonstrates that flood events pose a significant risk. The varying number of people affected, deaths, and damage to properties in different years highlights the unpredictable nature of floods and their potential consequences. While some years may experience low flood occurrences or even no reported incidents, other years show a substantial impact on communities, resulting in a high number of affected individuals, loss of life, and extensive damage to revenue estates and houses. This pattern underscores the importance of recognizing and preparing for the potential risks associated with floods, as they can have devastating effects on both human lives and infrastructure. Understanding the flood history serves as a reminder of the need for proactive measures, such as effective disaster management strategies and infrastructure planning, to mitigate the risks and protect vulnerable communities from future flood events.

**Fig 4:**
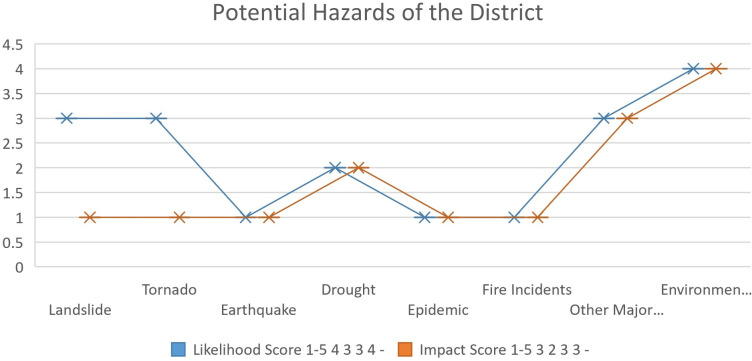
Potential hazards of the district Mianwali.

Fig 4 illustrates a comprehensive assessment of the risk associated with various hazards.

Among the hazards shown in Fig 3, floods, hill torrents, and environmental hazards were identified as high-risk (Supporting Information Tables 1–2 in S1 and S2 Files). Floods have a likelihood score of 4 and an impact score of 3, resulting in a risk score of 15–25. Similarly, hill torrents and environmental hazards also fall within the same risk score range, indicating a significant level of risk (Fig 4).

Urban flooding, flash floods, and other major accidents are considered as medium risk category. Urban flooding has a likelihood score of 3 and an impact score of 2, resulting in a risk score of 8–14. On the other hand, landslides, tornadoes, earthquakes, droughts, epidemics, fire incidents, and landslides are classified as low-risk hazards. These hazards have a risk score range of 1–7, indicating a relatively lower level of risk compared to the high and medium-risk hazards.

Overall, this assessment provides valuable insights into the varying levels of risk associated with different hazards. Such information is crucial for stakeholders to prioritize and allocate resources effectively to mitigate and manage these risks efficiently.

## Discussion

In this study, the results from the household study, the FGDs and key informant interviews on the impact of floods on people’s livelihoods are addressed with people’s perceptions of flooding as a threat to their livelihoods.

Climate extremes not only adversely affect livelihoods in the short term (in the form of yield losses, loss of human lives and productive assets, and disease spread), but can contribute to a series of long-term impacts such as increasing food prices and loss of agricultural productivity contributing to food shortages over the years [39].

The locals had shared their experiences that agriculture was a subsistence livelihood practice and it had become vulnerable due to dependence on water and temperature. Results of this study revealed that 52.5 percent of the households surveyed had no direct water source to irrigate the crops which may be count as a factor for their high vulnerability and sensitivity. It is extremely significant as agriculture is heavily dependent on water but infrastructure is either inadequate or unavailable to poor farmers [40].

As the pattern of rainfall has changed as told by local residents in FGD’s, the overall agricultural shift and productivity is thus disturbed. Local people noticed that as the rainfall was unpredictable, crop production was influenced by changes in the seeding and harvesting time [41]. Changing climate patterns have also influenced the livestock rearing, collection and selling of wood. Similar results were found in the Jumla district of Nepal where crop growth management activities were usual in both the front and back yard of homes [26].

Seasonal variation showed significant influence on their livelihood strategies particularly those related to the farming and fishing. It was found that 65% of the surveyed households had agriculture as the main livelihood. Most of the households with agriculture as their main earning source reported low agricultural productivity after the flood of 2010 which destroyed their land and damaged their crops. Floods and heavy rainfall wash away the growing field, which also contributes to a decline in crop production [42].

FGD participants had suggested that loss of crop and livestock is the most economically significant effect of flood. This is particularly alarming considering that the increasing frequency and severity of these disasters erode small landholders’ livelihoods, reducing their agricultural assets such as grains, seeds, and soil productivity, and magnifying their vulnerabilities. Moreover, because small farmers primarily meet their household food demand from crops, crop losses will intensify food insecurity [43].

The bulk of the households surveyed in that community was low to medium income and employed in the agriculture sector. Nearly half of the households surveyed were residing in highly vulnerable floodplains and some had also established houses near embankments. Such homes were unlawfully constructed within the floodplains when the floods had receded last time. The design and location of the residential buildings are known as the key factors in the vulnerability analysis [44]. During FGD citizens said their houses were constructed in a conventional fashion and ignored engineered steps. In the area of study there were three types of houses, i.e., i.e., Muddy, Partial Concrete, and Concrete. The research showed that most of the buildings were Muddy while others were Partially Concrete and very few were Concrete homes.

The results of this study revealed that, owing to flood damage, 47 per cent of families moved to other areas. As reported by Najam-u-Din [45], seven million residents around the country were prompted by the 2010 flooding to evacuate their homes at least temporarily. Hoq et al [46] reported similar findings that households in Sunamganj district were more vulnerable to flood hazard and natural disaster in terms of food, water, and health because of lowest adaptive capacity and highest sensitivity and exposure [46].People were confronted with problems due to the loss of houses and communication, health and education facilities, as well as reduced livelihood opportunities, at least in the short term, as the floods had massively destroyed crops and livestock [45]. Focus group discussion members and primary informants acknowledged that flooding has impacted livestock with elevated water quantity.

Well-off and medium-income respondents identified flood-affected farmlands and communal lands, such as forests and highways. Local residents also indicated that the trees were eradicated by the flood which leads to decreased supply of Non-Timber Forest Products (NTFPs). Several families engaged in collecting and marketing medicinal plants suffered when forestland was ruined by flood. Similar findings were found in Nepal when local people collecting fuel wood from nearby woods resulted in a reduced forest size [29].

Food and water vulnerabilities within the Mianwali district, as assessed by the LVI-IPCC, highlight critical challenges faced by the community. Food vulnerability is largely driven by the community’s dependence on agriculture, making it highly sensitive to climatic changes such as frequent flooding, which disrupts crop production and leads to food shortages. Economic factors, including low income and limited financial resources, hinder access to food during these crises, exacerbating food insecurity. Additionally, the resilience of local food systems plays a crucial role; when markets are disrupted, access to food diminishes, increasing vulnerability. Nutritional knowledge also affects dietary diversity and food choices, further contributing to malnutrition.

Water vulnerability focuses on the community’s ability to access clean and safe water, which is influenced by the management of water resources and the state of infrastructure. Ineffective management can lead to scarcity, especially during floods that overwhelm water systems, compromising water quality. Poor infrastructure exacerbates these issues, making it difficult for the community to access safe drinking water. Furthermore, changes in precipitation patterns can affect water availability, and flooding can lead to waterborne diseases when clean sources are contaminated.

The high exposure value (0.424) reflects significant risks from climatic events, directly impacting livelihoods, food security, and water accessibility. The overall vulnerability score (0.0305) underscores the interplay between low adaptive capacity and high exposure, indicating a pressing need for interventions to enhance resilience in food and water systems. Targeted strategies, such as improving agricultural practices, enhancing water management infrastructure, and increasing community awareness about nutrition and water safety, are essential to address these vulnerabilities effectively.

The results implied that adaptive capability for families should also be addressed explicitly, since community capacity is the secret to addressing economic, social and environmental issues. In addressing similar issues in Myanmar, Oo *et al*. [47] reported that lack of adaptive capability among households is a major cause of high vulnerability to the consequences of climate change and disasters.

It was reported that owing to inadequate knowledge and prevalent poverty, citizens had low adaptation capacity; as the locals’ coping mechanisms were the combination of adaptation action to adjust in changing climate. The lack of government support for the community is a big reason behind low adaptive ability. Just 6 percent of those surveyed received any form of social aid from private NGOs and 8.5 percent received assistance from government agencies. Only 39 percent of surveyed households mentioned having some form of warning the last time a flood occurred in the city.

It was also observed that the local people in research site were discontented with the health services accessible to them. Just 40 percent of the overall households surveyed were found to have basic (emergency) healthcare services. The presence of health care centers in their area was confirmed by 56.5 percent. Community members mentioned that most of the people were too far away during the floods and it was difficult for them to access the medical centers due to blockage of roads as roads and pathways were disrupted or ruined by water and the severity of the floods caused major loss of infrastructure [48].

Around 35.5 percent of the respondents claimed their family members suffered from chronic illness. In terms of sensitivity of livelihoods to flood impacts, human capital plays a fundamental role, especially if taken in terms of health. Health issues reduce labor capacities and result in a reduced number of working days [21].

Livelihood vulnerability was also analyzed according to gender of the household head. The findings of survey showed that the proportion of male- and female-headed households was 82.5% (n =  165) and 17.5% (n =  35), respectively. The findings of vulnerability study according to household head was relevant in the rural context of Nepal where number of female-headed households rapidly increased primarily due to migration of male members to the Gulf and other countries for labor and remittance purpose.

Adaptation includes steps and approaches to mitigate and combat the adverse effects of environmental disasters and climate change in the future [49]. The incidence of flood hazard impacts citizens in different ways. The individuals impacted were compelled to use coping strategies. The surveyed households adopted various preparedness and coping mechanism. 66% of the total households surveyed store food (grains, seeds) for use during or after flooding conditions. Food security improves resilience of households to external pressures like severe weather events [50–52].

## Conclusion

Mianwali District’s vulnerability, primarily due to its geographical location along the Indus River, has been exacerbated by severe flood events, notably the 2010 flood. This study utilized the Participatory Rural Appraisal (PRA) technique, including Focus Group Discussions (FGDs) and Transect Walks, to assess local community vulnerability, quantified through the Livelihood Vulnerability Index (LVI), which yielded a value of 0.4. This high LVI underscores the significant vulnerability of riverside communities, particularly driven by poverty and reliance on agriculture and forest resources.

To address these challenges, it is crucial for local government and community leaders to implement targeted rural development programs that enhance coping mechanisms and invest in resilient infrastructure. Specific recommendations include constructing embankments and flood shelters to mitigate flood impacts. Additionally, developing educational programs focused on flood awareness and preparedness will empower residents to respond effectively to hazards. Policymakers should also encourage economic diversification to reduce dependence on vulnerable agricultural and forest-based livelihoods.

Finally, ongoing research and monitoring are essential for understanding community resilience and developing adaptive strategies for flood mitigation. These measures are vital for alleviating the impacts of floods on vulnerable populations in Mianwali District and for promoting sustainable development in the region.

## Supporting information

S1 File Potential Hazards of Districts Mianwali. (XLSX)

S2 File Potential Hazards of Districts Mianwali per year. (XLSX)

S3 File Questionnaire: Adaptation Capacity of Local People to Climate Change. (DOCX)
