# Supplementary material for: Livelihood vulnerability index: Assessment of climatic changes in flood affected areas of Mianwali district, Punjab, Pakitan
Source: PLoS One. 2025 Mar 18;20(3):e0315398. doi: 10.1371/journal.pone.0315398 (PMC11918423; doi:10.1371/journal.pone.0315398)
Supplement: S3 File — (DOCX) [file pone.0315398.s003.docx]

**Supporting Information 3**

**Questionnaire**

**ADAPTATION CAPACITY OF LOCAL PEOPLE TO CLIMATE CHANGE**

“The purpose of this survey is to learn about the experiences of everyone regarding vulnerability capacity assessment and adaptation towards climate change. This survey is voluntary and confidential. That means that if you do not feel like answering the questions you do not have to, and if you feel like stopping part way through that is ok, you can stop at any time. It also means that your name will not be attached with your answers and [if appropriate] only the person interviewing you will know your answers.”

**Vulnerability capacity assessment in face of floods**

**General Information about community and respondent(s)**

| **To Be Filled By Interviewer** |
| --- |
| Date of Interview: **_________________________________**  Name Of village:__________________________________  Name of Tehsil:___________________________________  No. Of Household (for personal record):__________________ |

**Basic Information of respondents/ Households:**

Name of respondent:______________________

Gender:  Male/Female

Education:  no formal education         primary            secondary               higher

Head of household:     Male              Female

Type of household:  very poor            poor               better off                well off

Do you own land of your household:   Yes               No

Do you have access to electricity?    Yes                  No

Do you have access to Sui gas?      Yes                    No

Do you have access to clean drinking water?   Yes              No

Distance from your house to cemented road: ________________ minutes walking time

Migration pattern:    abroad            working in main district         working in same tehsil

N/A

Major source of income:    Agriculture           Forestry based             Jobs/ Services

Livestock based                Fishing based               combination of different sources

Is your income sufficient to cover and support your basic needs of life( Food, healthcare, clothing and schooling)?

Income is more than sufficient           less than sufficient             sufficient

Is there any income source or opportunity you no longer find( after flood)?

Over the period of last 10/20 years, is there any new opportunity or source of income in your area?

What are the important socio-economic changes noted in the region (after flood)?

**Resource Profiling Through Transect Walk**

What are the natural resources available for your daily activities?

What are the resources men usually use?

What is the purpose of use?

What are the resources women usually use?

What is the purpose of you?

What are the resources which were in your access before flood but no longer can access them? Name them (e.g fresh water, fuel wood, some plants as medicine).

What is the change in the abundance and seasonal availability of any resource?

How this change has affected your life (e.g loss of income source, unavailability of fuel wood)?

Any new resource available now which was not available previously (e.g plants or materials for energy production)?

**Interview questions for Focus Group Discussion (FGD)**

**Livelihood and Seasonal Mapping**

Number of floods in last 5 years?

Distance of village from the nearest river/canal/source of flood?

Who is the most vulnerable to the floods in your opinion?

How can flood influence the local community as a whole?

What type of a house do you live in?

Are there any physical structures that protect you from storms/floods?

Have you been directly impacted by the floods?

Have you been directly impacted by the floods?

When did it happen?

What were the impacts you or anyone in your household faced?

Have the flood affected your____________

Family                   land                 livestock            crops             any other

What was the impact of flood on the food availability of your household?

No change               less food                more food            uncertain

In a year, for how many months do you and your family has food storage?

0-3 months           3-6 months            6-9 months          9-12 months

How do households store food and water?

Are there any communal/government food storage capacity (Godowns)?

Is the access to food markets adequate (roads/markets)?

Do you have diversity of food in your area?

Grains available (rice, bread, cereals etc)                             sufficient vegetables

Sufficient dairy/meat or fish                                                  sufficient fruits

Have you ever felt any change in food diversity (after flood)?

Less                           more                         no change                uncertain

What are the main activities women/men carry out in your area?

How many members of your household are employed in work other than agriculture?

Do you own the land that you use for agriculture?

What is grown on land?

Is there any change in the crop productivity (after floods)?

Do you have any animal in your house (or livestock)?

Who takes care of livestock in the house?

Has the fish productivity been affected by the flood?

What are the migration patterns you do to pursue your life activities (daily, seasonal or yearly)?

Have members of the household migrated to other places for better paid income?

To what extent is agriculture dependent on rainwater?

Do girls have equal access to education?

Do people in your household have a balanced and healthy diet?

**Institutions**

Is there any disaster management committee in your area?

Are women represented in those communities?

Has the fish productivity been affected by the flood?

What are the migration patterns you do to pursue your life activities ( daily, seasonal or yearly)?

Have members of the household migrated to other places for better paid income?

To what extent is agriculture dependent on rainwater?

Do girls have equal access to education?

Do people in your household have a balanced and healthy diet?

**Institutions**

Is there any disaster management committee in your area?

Are women represented in these committees?

Is there any institution in the community for decision making/ management of resources e.g. forest, land, water bodies?

Are there any groups or arrangements in the community to help you resolve your problems?

Is there a provision of early warning systems with respect to flood?

**Coping strategies**

**(These questions should be answered after getting responses from seasonal and livelihood mapping)**

When there is more rainfall than expected, what you do with your crops?

When the weather is hotter than expected, what you do?

Your area faces floods mostly after rainfall, so how you deal with it?

During flood, what you do with your crops and livestock?

Have you introduced any new crop or left planting some old ones?

Do you receive any help from the community groups or any other institutions to overcome problems during and after floods?

**General Information**

1. Name of the Respondent :_____________________________
2. Age of the Respondent :

18-25 ( ) 26-35 ( ) 36-45 ( ) above 45 ( )

1. Gender :

Male ( ) female ( )

WHAT IS YOUR LEVEL OF EDUCATION?

- Middle
- Matriculation
- Intermediate
- Bachelors
- Masters

DO YOU KNOW THE PHENOMENA ABOUT CLIMATE CHANGE?

- Only heard
- Know a little about it
- Know a great deal about it

WHAT ARE THE WEATHER RELATED CHALLENGES YOU HAVE FACES?

- Unpredictable temperature
- Low temperature / High Temperature
- Unpredictable Precipitation
- Low Moisture/ High Moisture
- Strong winds/ Strong rainfall event
- Floods
- Short season
- Others

DO YOU FEEL ANY CHANGE IN CLIMATE REGARDING TEMPERATURE OVER THE PAST 10 YEARS?

YES ( ) NO ( )

DO YOU FEEL ANY CHANGE IN THE AMOUNT OF PRECIPITATION OVER THE PAST 10 YEARS?

YES ( ) NO ( )

WHAT ARE THE BARRIERS IN PROMOTING CLIMATE CHANGE AWARENESS?

- Lack of funds
- Lack of awareness
- Fear of Experiment
- Other

DOES MIGRATION IS A PART OF RESPONSE OF ADAPTIVE CAPACITY AGAINST FLOODS?

Yes ( ) No ( )

DOES THE POVERTY LEVEL AND LIVING CONDITIONS IMPROVED OR DETERIORATE IN MIANWALI AGAINST WEATHER CONDITIONS PARTICULARLY FOR FLOODS?

Yes ( ) No ( )

WHAT DO U THINK WHO HAS RESPONSIBILITY FOR INITIATING A RESPONSE TO THE IMPACTS OF CLIMATE CHANGE?

- Combination of Government and other
- Federal Government
- Municipal Government
- NGOs
- No response needed

WHAT PHASE OF ADAPTATION YOU ARE CURRENTLY WORKING ON?

- Currently not involved
- Phase of Understanding
- Phase of Planning
- Phase of Implementation

ARE YOU CONSERVING WATER AND SOIL?

Yes ( ) No ( )

ARE YOU USING ORGANIC MANURES?

Yes ( ) No ( )

ARE YOU USING INORGANIC FERTILIZERS?

Yes ( ) No ( )

ARE YOU USING THE STRATEGY OF COVER CROPPING?

Yes ( ) No ( )

ARE YOU USING MINIMUM TILLAGE SYSTEM?

Yes ( ) No ( )

ARE YOU USING IRRIGATION SYSTEM?

Yes ( ) No ( )

DO YOU LISTEN TO INFORMATION REGARDING CLIMATE CHANGE?

Yes ( ) No ( )

DO YOU CHANGE THE DATES OF HARVESTING?

Yes ( ) No ( )

DO YOU DO PLANTING OF CROPS WITH EARLY RAINFALL?

Yes ( ) No ( )

DO YOU USE PEST AND DISEASE RESISTANT CROPS?

Yes ( ) No ( )

DO YOU USE WINDBREAKS/ SHELTER BELTS?

Yes ( ) No ( )

DO YOU PARTICIPATE IN RECLAMATION PROCESSES TO WETLANDS?

Yes ( ) No ( )

WHAT STRATEGY YOU ARE USING FOR THE CROPS AGAINST THE CLIMATE CHANGE?

- Diversification of crops
- Short Duration Variety
- Hybrid Variety
- Organic Farming

WHAT IS THE REASON BEHIND CHANGING PATTERNS OF CROPS?

Climate change ( ) Other ( ) Don’t Know ( )

ARE YOU USING ANY SOIL MANAGEMENT PRACTICES?

Yes ( ) No ( )

WHAT PRACTICES YOU ARE USING FOR SOIL MANAGEMENT FOR WEATHER RELATED CHALLENGES?

- Conservation Tillage
- Covering the crops
- Crop Rotations
- Organic Fertilizers

HOW WELL LOCALS ARE EQUIPPED WITH CAPACITIES (KNOWLEDGE AND EXPERIENCE) FOR CLIMATE CHANGE ADAPTATIONS?

- 10-30 %
- 30-50 %
- 50-70%
- >70 %

TO WHAT EXTENT DO PARTNERSHIPS EXIST BETWEEN COMMUNITIES, PRIVATE SECTOR AND LOCAL AUTHORITIES TO REDUCE RISK OF CLIMATE CHANGE?

- 35 %
- 50%
- More than 75 %
- Lack of Knowledge

HOW FAR DO LAND USE POLICIES AND PLANNING REGULATIONS FOR HOUSING AND DEVELOPMENT INFRASTRUCTURE TAKE CURRENT AND PROJECTED CLIMATE RELATED RISKS INTO ACCOUNT?

Housing Yes ( ) No ( )

Communication Yes ( ) No ( )

Transportation Yes ( ) No ( )

Energy Yes ( ) No ( )

DOES THE LOCAL GOVERNMENT CONDUCT AWARENESS OR EDUCATION PROGRAMS ON DISASTER PREPAREDNESS FOR LOCAL COMMUNITIES?

Yes ( ) No ( )
